# Relationship Between Clozapine-Induced Inflammation and Eosinophilia: A Retrospective Cohort Study

**DOI:** 10.1093/schbul/sbae213

**Published:** 2024-12-16

**Authors:** Yuki Kikuchi, Yuji Otsuka, Fumiaki Ito, Yuji Yada, Hiroaki Tanifuji, Hiroshi Komatsu, Hiroaki Tomita

**Affiliations:** Department of Psychiatry, Graduate School of Medicine, Tohoku University, Sendai, Miyagi, 980-8574, Japan; Department of Psychiatry, Kodama Hospital, Ishinomaki, Miyagi, 986-0873, Japan; Department of Psychiatry, Asahi General Hospital, Asahi, 289-2511, Japan; National Hospital Organization Hanamaki Hospital, Hanamaki, 025-0033, Japan; Department of Psychiatry, Okayama Psychiatric Medical Center, Okayama, 700-0915, Japan; Department of Pharmacy, Kodama Hospital, Ishinomaki, Miyagi, 986-0873, Japan; Department of Psychiatry, Tohoku University Hospital, Sendai, Miyagi, 980-8574, Japan; Department of Psychiatry, Graduate School of Medicine, Tohoku University, Sendai, Miyagi, 980-8574, Japan; Department of Psychiatry, Tohoku University Hospital, Sendai, Miyagi, 980-8574, Japan

**Keywords:** C-reactive protein, DRESS, fever, myocarditis, pneumonia

## Abstract

**Background and Hypothesis:**

Eosinophilia has not been highlighted in clozapine-induced adverse inflammatory events, as it is often asymptomatic and self-limiting, while drug reaction with eosinophilia and systemic symptoms (DRESS) syndrome occurs rarely. This study aimed to reveal the temporal relationships between eosinophilia and other inflammatory events during clozapine initiation.

**Study Design:**

The temporal relationships between eosinophilia and other inflammatory events were evaluated among 241 patients with schizophrenia treated with clozapine for the first time at 7 hospitals. Risk factors for eosinophilia were investigated among preceding inflammatory events and other clinical characteristics. Furthermore, patients with eosinophilia were stratified by the severity of adverse inflammatory events and their clinical characteristics were compared.

**Study Results:**

Of the 54 patients who experienced inflammatory adverse events, 27 (50%) developed eosinophilia. In all but 1 patient, clinical symptoms of inflammatory adverse events preceded eosinophilia. In contrast, of the 187 patients without inflammatory events, 21 (11%) developed eosinophilia. Multivariate analysis revealed that more severe preceding inflammatory adverse events were associated with a greater risk of eosinophilia. The median time to the first detection of eosinophilia and peak eosinophil count occurred significantly earlier in patients with severe adverse events than in asymptomatic patients.

**Conclusions:**

In most cases, eosinophilia developed after the onset of inflammatory symptoms. Preceding inflammation was associated with the development of clozapine-induced eosinophilia. Eosinophilia may not be suitable as an early detection marker of severe inflammatory adverse effects. These findings enhanced our understanding of the involvement of eosinophilia in clozapine-induced inflammatory events.

## Introduction

Clozapine-induced eosinophilia is a relatively common and generally self-limiting side effect.^1,2^ Eosinophilia has been observed in 13%-14% of clozapine-treated patients based on a threshold of ≥400/mm³,^3,4^ and the 1-year cumulative incidence of eosinophilia with a threshold of 1500/mm³ was 5.9%.^5^ In our previous research, we observed that approximately 20% of Japanese patients experienced eosinophilia based on the threshold of ≥500/mm³ during clozapine titration.^6^ Eosinophilia typically occurs within 2-5 weeks after initiating clozapine, resolving spontaneously within 4 weeks without requiring drug discontinuation,^3,4^ and it is generally dose-independent.^7^ While clozapine-induced eosinophilia can be associated with severe inflammatory side effects causing organ damage, such as myocarditis and nephritis, it is more often asymptomatic and self-limiting.

Similar to eosinophilia, clozapine-induced fever is also common.^1,2^ Fever raises clinical concern as a potential indicator of more severe side effects, such as myocarditis. If fever occurs during clozapine administration, dose reduction or discontinuation of the prescription may be considered. In contrast, clozapine-induced eosinophilia has received less clinical attention, as it is typically asymptomatic and self-limiting. There have been reports of drug reaction with eosinophilia and systemic symptoms (DRESS) syndrome caused by clozapine resulting in severe organ damage. However, they are recognized as rare events.^8–11^ For this reason, little attention has been paid to the relationship between eosinophilia and the mild to severe inflammatory side effects that occur during clozapine administration. The primary aim of this study was to confirm whether inflammatory adverse events precede eosinophilia, and to verify whether more severe preceding inflammatory adverse events increase the risk of eosinophilia compared to asymptomatic patients. Additionally, we aimed to explore whether eosinophilia could serve as an indicator for severe inflammatory adverse events. The secondary objective was to examine differences in eosinophilia onset and values among 3 patient groups: asymptomatic patients, those with fever but no severe organ damage, and those with severe organ damage.

## Methods

### Participants

This study conducted a secondary analysis of a previously collected data set.^6,12,13^ We retrospectively reviewed the medical records of all 272 patients with treatment-resistant schizophrenia who initiated clozapine for the first time at 7 hospitals between July 2009 and February 2023. Participating centers included Tohoku University Hospital, Kodama Hospital, Miyagi Psychiatric Center, Hanamaki Hospital, Kunimidai Hospital, Aoba Hospital, and Asahi General Hospital. Diagnoses of schizophrenia based on the 10th Revision of the International Classification of Diseases (ICD-10) were obtained from medical records. ICD-10 is widely used in Japan for official statistics, official medical certificates, and medical record management at medical institutions. Treatment resistance was defined as the failure to achieve a Global Assessment of Functioning score ≥41 points following treatment with at least 2 antipsychotics (chlorpromazine equivalent ≥600 mg) for a minimum of 4 weeks, following the definition provided with the Japanese clozapine package insert.

Of the 272 initial cases, 31 were excluded due to specific criteria: 8 cases involved medication refusal or consent withdrawal, 4 cases were discontinued due to unrelated physical complications, 2 cases were discontinued due to abnormal laboratory values within 1 week of clozapine initiation, and 17 cases lacked essential data. The final analysis included 241 cases.

### Study Design

First, we examined whether inflammatory adverse effects preceded eosinophilia in patients who had both inflammatory adverse effects and eosinophilia. Data collected from medical records for 90 days from the start of clozapine treatment included the following: age at clozapine initiation, sex, body mass index (BMI), smoking status, concomitant use of valproic acid, clozapine initiation date, daily clozapine dose, onset date of clinical symptoms including fever, C-reactive protein (CRP) levels and measurement dates, eosinophil count and measurement dates, and detailed information regarding clozapine-induced inflammatory adverse effects. The reference range for eosinophil count is 0-500/mm^3^.^14^ Eosinophilia is defined as an eosinophil count >500/mm^3^, and hypereosinophilia is defined as an eosinophil count >1500/mm^3^.^14^ In this study, the cutoff of eosinophilia was set at 600/mm³, lower than the cutoff of hypereosinophilia (>1500/mm³), because it was necessary to clarify the relationship between eosinophilia and inflammatory adverse effects by including the milder to the pronounced levels of eosinophilia. We confirmed that none of the 241 patients in this study had medical comorbidities that could cause an increase in eosinophil count such as asthma or atopic dermatitis, and that none of the patients had a persistently high eosinophil count of >600/mm^3^. The eosinophil levels were measured concurrently with mandatory neutrophil count monitoring, conducted weekly. In cases where neutrophil counts were required twice a week or where clinicians ordered additional tests based on the patient’s condition, eosinophil counts were measured more frequently. Inflammatory adverse effects were defined as follows: (1) fever, (2) low-grade fever, and (3) clinical symptoms necessitating the discontinuation of clozapine or dose reduction. In Japan, hospitalization for 18 weeks is required when initiating clozapine treatment. Therefore, patients’ body temperature and clinical symptoms were monitored daily. Fever was defined as an axillary temperature of 38 °C or higher, while low-grade fever was defined as a temperature between 37 °C and 38 °C. Participants who had inflammatory adverse effects that resulted in being diagnosed as myocarditis, pneumonia, renal failure, liver damage, or skin rashes were classified into a group of severe adverse events (severe organ damage). Myocarditis was diagnosed by a cardiologist based on findings such as elevated troponin and brain natriuretic peptide levels, abnormal electrocardiograms, and echocardiograms. Pneumonia was diagnosed using chest radiographs or computed tomography scans. Renal failure was diagnosed based on an increase in serum creatinine level from baseline. Liver damage was diagnosed based on an increase in aspartate aminotransferase and alanine aminotransferase levels from baseline. Skin rashes referred to rashes that occur all over the body, and were diagnosed by visual observation. The clozapine titration rate (CTR) was calculated using the manufacturer-recommended protocol (increasing the dose by 25 mg/day every few days up to 200 mg/day in 3 weeks) as a reference (CTR = 1). Patients were classified into the faster titration group if their CTR was greater than 0.75, and into the slower titration group if their CTR was less than 0.75, based on international guidelines recommending a CTR of approximately 0.75 for Asian patients to prevent inflammatory adverse effects.^15^

Second, we analyzed risk factors for eosinophilia using a case-control design, categorizing patients with eosinophilia as cases and those without eosinophilia as controls. Risk factors included the severity of preceding inflammation (asymptomatic, nonsevere [fever without severe organ damage], and severe [with severe organ damage] groups), sex, obesity (BMI > 30 kg/m^2^), and concomitant use of valproic acid.

Third, we stratified patients with eosinophilia into 3 groups according to the severity of preceding inflammation: asymptomatic, nonsevere (fever without severe organ damage), and severe (with severe organ damage). Then, we analyzed differences in patient characteristics across these groups.

### Statistical Analysis

Statistical analyses were performed using EZR software (Jichi Medical University). Differences in demographic data between cases and controls were analyzed using Fisher’s exact test for categorical variables and *t*-tests or Mann-Whitney *U* tests for continuous variables. Analysis of variance or the Kruskal-Wallis test was used to compare continuous variables among the 3 groups. Logistic regression analysis was used to categorize the patients into 3 groups according to the categorical variable “inflammatory adverse events”: the asymptomatic, nonsevere, and severe groups. Inflammatory adverse events (asymptomatic, nonsevere, severe), sex, obesity status (BMI > 30 kg/m^2^), and concomitant use of valproate were selected as variables influencing the risk of eosinophilia and were evaluated using logistic regression analysis. We also conducted a logistic regression analysis in which being overweight (BMI > 25 kg/m^2^) was added as an explanatory factor instead of obesity as a sensitivity analysis. Additionally, multiple regression analysis was performed to assess variables affecting the first detection date of eosinophilia and the date of peak eosinophil count. This study was approved by the Ethics Committee of the Tohoku University Graduate School of Medicine (Approval ID: 2022-1-1136).

## Results

### Relationship Between Onset Date of Inflammatory Adverse Events and Onset of Eosinophilia

**Figure 1.**
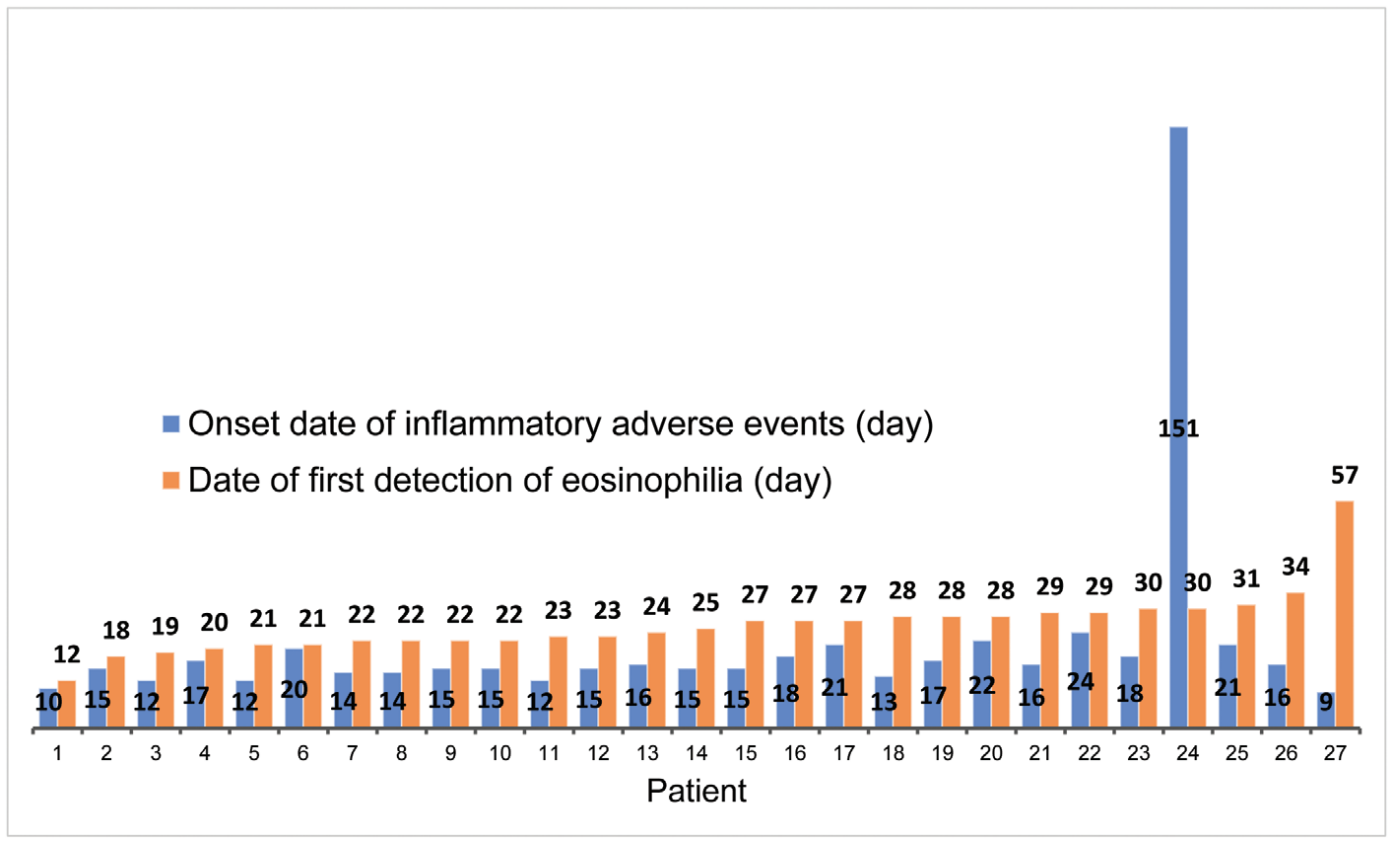
Relationship between the onset date of inflammatory adverse events and the onset date of the first detection of eosinophilia in 27 patients with both eosinophilia and inflammatory adverse events.

**Figure 2.**
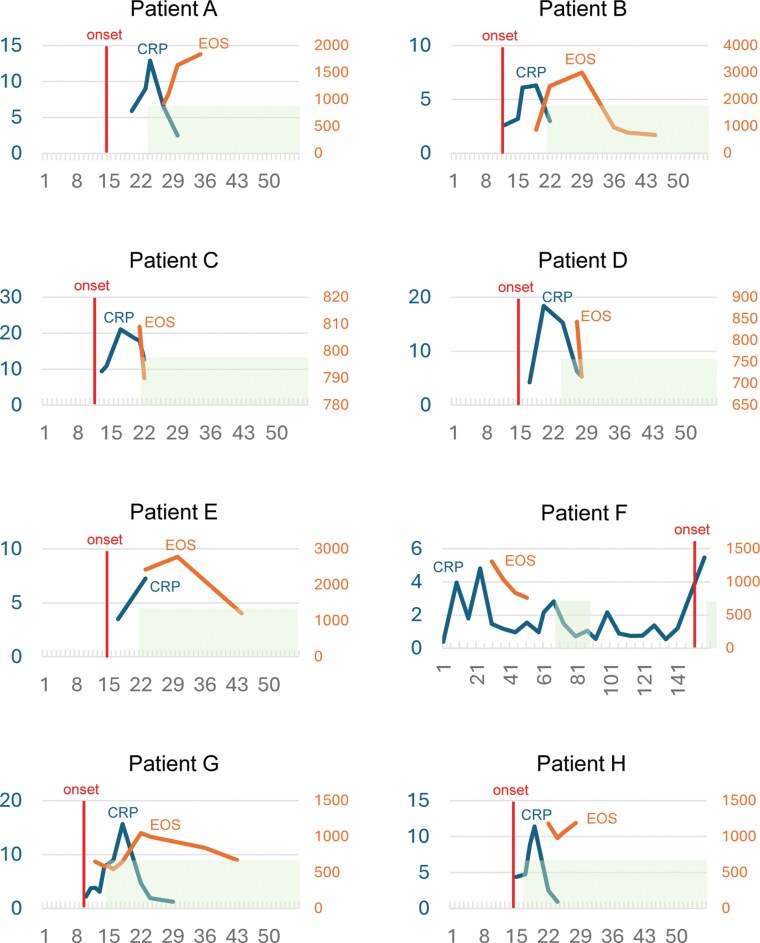
Time course of 8 patients who experienced severe adverse reactions associated with eosinophilia, leading to the discontinuation of clozapine. The left axis represents C-reactive protein (CRP) levels (mg/dL), while the right axis indicates eosinophil count (EOS; /mm³). The horizontal axis shows the number of days since the initiation of clozapine treatment. The “Onset” marker denotes the date when the patient first developed clinical symptoms. Shaded areas indicate the period after clozapine discontinuation.

Of the 54 patients who experienced inflammatory adverse events, 27 (50%) had eosinophilia. In contrast, of the 187 patients who did not experience inflammatory adverse events, 21 (11%) had eosinophilia. The relationship between the onset date of inflammatory adverse events and the onset date of the first detection of eosinophilia was examined in 27 patients who experienced both eosinophilia and inflammatory adverse events (Figure 1). In all but 1 patient, clinical manifestations of inflammatory adverse effects preceded the onset of eosinophilia. Figure 2 shows the time course of 8 patients who experienced severe adverse reactions associated with eosinophilia that led to the discontinuation of the drug, indicating that inflammation preceded eosinophilia.

### Comparison of Patients With and Without Eosinophilia

Next, we conducted a case-control study to verify whether the severity of inflammatory adverse events is a risk factor for eosinophilia. There were no significant differences between patients with and without eosinophilia in terms of age, sex, obesity status, titration group, concomitant use of valproic acid, or smoking status (Table 1). All inflammatory adverse events were significantly more frequent in patients with eosinophilia (27/48 [56%] vs 27/194 [14%]; *P* < .001). Severe adverse events were also significantly more common among patients with eosinophilia (10/48 [21%] vs 4/194 [2.1%]; *P* < .001). Furthermore, fever duration was significantly longer in patients with eosinophilia. Although maximum body temperature and peak CRP levels tended to be higher in patients with eosinophilia, these differences were not statistically significant.

**Table 1.** Comparison of Patients With and Without Eosinophilia

	Patients with eosinophilia	Patients without eosinophilia	Statistical test	*P*
Number of patients, *n*	48	193		
Number of patients with hypereosinophilia (eosinophil count >1500/mm^3^), *n* (%)	15 (31)	NA		
Age, years, mean (SD)	39.6 (11.1)	41.4 (13.1)	*t* = 0.86, df = 239	.39
Male, *n* (%)	27 (56)	108 (56)	Fisher’s exact test	1
BMI, kg/m^2^, mean (SD)	24.7 (5.5)	24.2 (5.0)	*t* = −0.61, df = 239	.54
Being overweight (BMI > 25 kg/m^2^), *n* (%)	16 (34)	72 (37)	Fisher’s exact test	.74
Obesity (BMI > 30 kg/m^2^), *n* (%)	9 (19)	17 (8.8)	Fisher’s exact test	.066
Faster titration group, *n* (%)	28 (58)	82 (42)	Fisher’s exact test	.053
Concomitant use of valproate, *n* (%)	13 (27)	43 (22)	Fisher’s exact test	.57
Smoking, *n* (%)	6 (13)	26 (14)	Fisher’s exact test	1
All inflammatory adverse events, *n* (%)	27 (56)	27 (14)	Fisher’s exact test	<.001
Severe adverse events, *n* (%)	10 (21)	4 (2.1)	Fisher’s exact test	<.001
Maximum body temperature, °C, median (IQR)	39.1 (38.6-39.5)^a^	38.6 (38.0-39.1)^b^	Mann-Whitney *U* test	.098
Peak CRP, mg/dL, median (IQR)	6.1 (2.5-14.3)^c^	4.3 (1.9-7.9)	Mann-Whitney *U* test	.11
Date of peak CRP, day, median (IQR)	19 (17-23.5)^c^	20.5 (18-23.5)	Mann-Whitney *U* test	.53
Fever duration, day, median (IQR)	7 (4-10)^a^	4 (2-5)^b^	Mann-Whitney *U* test	.0082
Peak eosinophil count, /mm^3^, median (IQR)	1023 (715-1791)	NA		
Date of peak eosinophil count, days, median (IQR)	29 (27-37)	NA		
Duration of eosinophilia, week, median (IQR)	2 (1-3)	NA		

Abbreviations: BMI = body mass index; CRP = C-reactive protein; IQR = interquartile range; NA = not applicable; SD = standard deviation.

^a^ *n* = 24.

^b^ *n* = 25.

^c^ *n* = 26.

### Logistic Regression Analysis for the Risk of Clozapine-Induced Eosinophilia

Multivariate analysis revealed that preceding adverse inflammatory events significantly increased the risk of eosinophilia (Table 2). The risk of eosinophilia was significantly higher in patients with nonsevere inflammatory adverse events (odds ratio [OR] 6.15; 95% confidence interval [CI] 2.80-13.5; *P* < .001) and in those with severe adverse events (OR 28.4; 95% CI 6.90-116; *P* < .001) than in those without inflammatory adverse events. In this analysis, sex, obesity status, and concomitant use of valproate were not significant risk factors for eosinophilia. As the standard measure of obesity in Japan is a BMI > 25 kg/m^2^, we also conducted a logistic regression analysis using being overweight (BMI > 25 kg/m^2^) as an explanatory factor in the sensitivity analysis (Supplementary Table S1). As a result, the overweight OR was 0.90, lower than the obesity OR of 2.29.

**Table 2.** Logistic Regression Analysis for the Risk of Clozapine-Induced Eosinophilia

	OR	95% CI	*P*
Inflammatory adverse events (nonsevere)^a^	6.15	2.80-13.5	<.001
Inflammatory adverse events (severe)^a^	28.4	6.90-116	<.001
Sex (male)	0.89	0.42-1.86	.75
Obesity	2.29	0.83-6.28	.11
Concomitant valproate	0.61	0.23-1.60	.31

Abbreviations: CI = confidence interval; OR = odds ratio.

^a^The asymptomatic group is the reference category.

### Comparison Between 3 Groups of Patients With Eosinophilia and Different Severities of Inflammatory Adverse Effects

Patients with eosinophilia were stratified into 3 groups based on the severity of preceding inflammatory adverse events: the asymptomatic, nonsevere, and severe groups, and the characteristics of treatment history and clinical manifestations were compared among the 3 groups (Table 3). The proportion of patients with faster titration or concomitant valproate use differed significantly among the 3 groups. The median date of the first detection of eosinophilia was significantly earlier in the severe group than in the asymptomatic group (22.5 days vs 31 days; *P* < .05). Similarly, the median peak eosinophil count occurred significantly earlier in the severe group than in the asymptomatic group (28 days vs 40 days; *P* < .05). The median peak eosinophil count was also significantly earlier in the nonsevere group than in the asymptomatic group (29 days vs 40 days; *P* < .05). These results remained robust after adjusting for the clozapine titration speed and concomitant valproic acid use in a multivariate analysis (Supplementary Tables S2 and S3). On comparing the severe and nonsevere groups, no significant differences in the onset date of preceding inflammatory adverse events, the date of first detection of eosinophilia, or the date of peak eosinophil count were noted. Peak eosinophil count and duration of eosinophilia were not significantly different among the 3 groups.

**Table 3.** Comparison of 3 Groups of Patients With Eosinophilia and Different Severities of Inflammatory Adverse Effects

	Severe group	Nonsevere group	Asymptomatic group	Statistical test	*P*
Number of patients, *n*	10	18	20		
Number of patients with hypereosinophilia (eosinophil count >1500/mm^3^), *n* (%)	4 (40)	6 (33)	5 (25)	Fisher’s exact test	.73
Age, years, mean (SD)	39.6 (7.73)	41.7 (11.0)	37.8 (12.6)	*F*(2, 45) = 0.60	.55
Male, *n* (%)	9 (90)	8 (44)	10 (50)	Fisher’s exact test	.053
BMI, kg/m^2^, mean (SD)	25.1 (5.0)	24.9 (5.1)	24.4 (6.3)	*F*(2, 45) = 0.068	.94
Being overweight (BMI > 25 kg/m^2^), *n* (%)	3 (30)	7 (39)	8 (40)	Fisher’s exact test	.86
Obesity (BMI > 30 kg/m^2^), *n* (%)	2 (20)	4 (22)	3 (15)	Fisher’s exact test	.89
Faster titration group, *n* (%)	9 (90)	14 (78)	5 (25)	Fisher’s exact test	<.001
Concomitant use of valproate, *n* (%)	7 (70)	3 (17)	3 (15)	Fisher’s exact test	.0043
Smoking, *n* (%)	0 (0)	3 (17)	3 (15)	Fisher’s exact test	.63
Onset date of inflammatory adverse events, day, median (IQR)	15 (12-18)	16 (14-18)	NA	Mann-Whitney *U* test	.64
Peak CRP level, mg/dL, median (IQR)	11.4 (6.1-15.7)^a^	5.6 (2.5-15.0)^b^	1.2 (1.0-1.4)^c^	Kruskal-Wallis test	<.001
Date of first detection of eosinophilia, day, median (IQR)	22.5 (20-27)	26 (22-29)	31 (29-42)	Kruskal-Wallis test	.0083^d^
Date of peak eosinophil count, days, median (IQR)	28 (27-29)	29 (25.5-34)	40 (32-47)	Kruskal-Wallis test	.0015^e^
Peak eosinophil count, /mm^3^, median (IQR)	1249 (895-2545)	1144 (896-1862)	921 (713-1539)	Kruskal-Wallis test	.30
Duration of eosinophilia, week, median (IQR)	2 (1-3)	1 (1-3)	2 (1-4)	Kruskal-Wallis test	.20

Abbreviations: BMI = body mass index; CRP, C-reactive protein; IQR = interquartile range; NA = not applicable; SD = standard deviation.

^a^ *n* = 9.

^b^ *n* = 17.

^c^ *n* = 5.

^d^Severe group vs nonsevere group, *P* = .49; severe group vs asymptomatic group, *P* = .020; nonsevere group vs asymptomatic group, *P* = .094 (all *P*-values are after Bonferroni adjustment).

^e^Severe group vs nonsevere group, *P* = 1; severe group vs asymptomatic group, *P* = .0048; nonsevere group vs asymptomatic group, *P* = .0167 (all *P*-values are after Bonferroni adjustment).

Supplementary Table S4 shows the characteristics of 8 patients who experienced severe eosinophilia-related adverse effects that led to clozapine discontinuation. In 7 patients, the onset of physical symptoms, including fever, preceded the detection of eosinophilia, except for patient F (Figure 2; Supplementary Table S4). Eosinophilia was first detected within 30 days in all 8 patients, and in 7 of these patients, the peak eosinophil counts also occurred within 30 days. In 4 of the 8 patients, clozapine was discontinued before eosinophilia was detected. Supplementary Figure S1 illustrates the trends of eosinophil counts in 17 of 21 patients without adverse inflammatory effects (excluding 4 patients in whom eosinophilia was detected at only 1 time point). Compared to the 8 patients with severe adverse effects, the timing of the first appearance of eosinophilia varied: 9 patients had their first detection after 30 days, and 16 patients had a peak eosinophil count after 30 days.

## Discussion

To our knowledge, this is the first study to demonstrate an association between clozapine-induced inflammation and eosinophilia. First, we observed that inflammatory adverse effects preceded eosinophilia in almost all patients. Therefore, we found that the presence of inflammation significantly increases the subsequent risk of eosinophilia. Furthermore, multivariate analysis revealed that patients with severe adverse effects had a higher OR for the risk of eosinophilia than those with nonsevere adverse effects. Therefore, the results suggest that the risk of eosinophilia increased with the severity of the preceding inflammation. Our results strongly suggest that eosinophilia is induced by clozapine-related inflammation. Moreover, the onset and peak of eosinophilia occurred significantly earlier in patients who developed severe adverse events than in asymptomatic patients. The delayed onset of eosinophilia in asymptomatic patients suggests that milder preceding inflammation (ie, the more asymptomatic the patient) is associated with a lower risk of eosinophilia and a later onset.

The fact that clinical symptoms and inflammation preceded eosinophilia in almost all patients in this cohort indicates that eosinophilia may not be suitable as an early detection marker of severe adverse effects. This observation aligns with previous studies showing that CRP and troponin levels are more valuable indicators for detecting clozapine-induced myocarditis than eosinophilia.^16^ Additionally, the lack of significant differences in the onset of clinical symptoms and eosinophilia between the severe and nonsevere groups of patients with eosinophilia suggests that eosinophil count cannot be a stand-alone marker for predicting the severity of inflammation among patients with inflammatory symptoms. Nevertheless, the findings might suggest that monitoring eosinophil count along with other inflammatory signs might help evaluate the risk of severe inflammatory adverse events during clozapine titration. Further research is needed to elucidate the mechanism underlying the involvement of eosinophilia in clozapine-induced inflammatory adverse events and to apply the knowledge to clinical use. In contrast, the clinical utility of CRP monitoring during clozapine titration to prevent clozapine-induced inflammation is well-established,^15,17,18^ with elevated CRP levels potentially predicting subsequent eosinophilia.

Slower clozapine titration has been reported to reduce the risk of inflammatory adverse events.^6,19^ In Japanese individuals, clozapine metabolism capacity is lower than in other ethnic groups; therefore, the guidelines recommend the slowest titration rate.^15^ In our previous research in Japanese individuals, a titration rate of 25 mg or less per week was safer.^6,19^ In the cohort of the current study, 46% (110/241) of patients underwent faster titration than the titration speed recommended by the guidelines. As a result, the incidence of inflammatory adverse reactions was high (22% [54/241]), and therefore, the incidence of eosinophilia was also high (20% [48/241]). Therefore, gradual titration may help alleviate inflammation and reduce the incidence of eosinophilia. Extrapolating from these results, in other ethnic groups, faster titration speeds than those recommended in the guidelines may increase inflammatory adverse effects and eosinophilia.

Although multivariate analysis did not show obesity to be a significant risk factor for eosinophilia, the OR decreased in the overweight group in the sensitivity analysis. Accordingly, it is possible that obesity was not detected as a statistically significant risk factor due to the small sample size. Further research is needed to determine the risk of obesity in the inflammatory side effects of clozapine.

While this study emphasizes the link between inflammation and eosinophilia, most cases of asymptomatic clozapine-induced eosinophilia are benign and resolve independently. All 20 asymptomatic eosinophilia cases in this cohort continued clozapine without any issues (Supplementary Figure S1). However, among the cohort, 10 patients with eosinophilia exhibited severe organ damage, with 8 meeting the criteria for possible DRESS syndrome.^6,20^ Thus, patients who develop eosinophilia during clozapine titration should undergo careful monitoring for symptoms of organ damage. Nearly all inflammatory adverse events in our cohort occurred in the early phase of clozapine titration, with eosinophilia developing in the later phase. This pattern has been observed in many previous reports of DRESS syndrome.^8–11^ Patient F in the current study was the only case in our cohort with a different progression pattern; eosinophilia appeared without fever, followed by a delayed onset of skin rash and pneumonia. There have been 2 prior case reports where eosinophilia preceded clinical symptoms. In the first case,^21^ eosinophilia was detected on day 29 (2575/mm^3^), followed by low-grade fever and myalgia on day 35, which progressed to pleural effusion, hepatitis, and jaundice. In the second case,^22^ eosinophilia appeared and persisted from 1 month after clozapine initiation, with the patient later developing a cough on day 122, leading to the observation of eosinophilic pleural effusion. Patient F and these case reports illustrate a pattern of asymptomatic eosinophilia followed by the gradual onset of clinical symptoms. DRESS syndrome can also develop during the maintenance phase.^8,11^ Thus, clinicians should be aware of syndromes that may gradually arise following eosinophilia.

This study had several limitations. First, eosinophil counts were measured through mandatory weekly neutrophil count blood sampling, which may have missed fluctuations in eosinophil counts within the week. In most cases in this cohort, eosinophilia lasted for 2 weeks or more, but it is possible that some cases of mild eosinophilia lasting less than 1 week were missed and eosinophilia was underestimated. Second, although clinical symptoms, such as fever, were monitored daily during hospitalization, the CRP levels were only measured at the clinicians’ discretion, leaving asymptomatic patients without CRP data. However, since 2023, we have been measuring CRP levels weekly during clozapine titration but this was not performed for patients in this cohort. In the future, we would like to examine the relationship between weekly CRP levels and the development of fever and eosinophilia, including asymptomatic patients. Third, the effects of concomitant medications other than valproic acid, such as lithium, carbamazepine, and other antipsychotics, were not investigated. Fourth, this study focused on the first 90 days after clozapine initiation, so clinical symptoms occurring beyond this period were not investigated. Fifth, this study was conducted exclusively in Japanese patients, which may limit the generalizability of the findings to other ethnic groups. Sixth, this study observed that inflammation often precedes eosinophilia, but the causal relationship is still speculative. Previous studies have suggested that clozapine alters the immune system and cytokine profiles of patients with schizophrenia, which may be related to the therapeutic and side effects of clozapine.^23^ Clozapine may have anti-inflammatory and pro-inflammatory effects,^23^ but these have not been fully understood. At least, cytokines such as interleukin (IL)-6 and tumor necrosis factor-α are elevated in the early titration period.^17,23^ To clarify these relationships, further studies should explore biological mechanisms, such as cytokines such as IL-5, related to eosinophilia.

In conclusion, preceding inflammation was associated with the development of clozapine-induced eosinophilia. In most cases, eosinophilia followed the occurrence of inflammatory symptoms. Eosinophilia may not be suitable as an early detection marker of severe inflammatory adverse effects. The findings added knowledge regarding the involvement of eosinophilia in clozapine-induced inflammatory events.

## Supplementary Material

sbae213_suppl_Supplementary_Tables

sbae213_suppl_Supplementary_Figure_S1

## Data Availability

The data are not publicly available because they contain information that could compromise participants’ privacy/consent.
