# Supplementary material for: Relationship Between Clozapine-Induced Inflammation and Eosinophilia: A Retrospective Cohort Study
Source: Schizophr Bull. 2024 Dec 16;52(1):sbae213. doi: 10.1093/schbul/sbae213 (PMC12809819; doi:10.1093/schbul/sbae213)
Supplement: sbae213_suppl_Supplementary_Tables [file sbae213_suppl_supplementary_tables.docx]

| Supplementary Table 1: Logistic regression analysis for the risk of clozapine-induced eosinophilia (Sensitivity analysis for being overweight) | | | |
| --- | --- | --- | --- |
|  | OR | 95% CI | P |
| Inflammatory adverse events (non-severe)^a^ | 6.43 | 2.94–14.1 | < 0.001 |
| Inflammatory adverse events (severe)^a^ | 26.3 | 6.46–107 | < 0.001 |
| Sex (male) | 0.87 | 0.41–1.77 | 0.71 |
| Overweight (BMI >25 kg/m^2^) | 0.90 | 0.43–1.91 | 0.79 |
| Concomitant valproate use | 0.70 | 0.28–1.77 | 0.45 |
| a: The asymptomatic group is the reference category. | |  |  |
| **Abbreviations:** CI, confidence interval; OR, odds ratio; BMI, body mass index |  |  |  |

| Supplementary Table 2: Multivariate analysis for the first detection date of eosinophilia | | | |  |  |
| --- | --- | --- | --- | --- | --- |
|  | Estimate | SE | 95% CI | T | P |
| Inflammatory adverse events (non-severe)^a^ | -5.21 | 3.52 | -12.3 to 1.89 | -1.48 | 0.15 |
| Inflammatory adverse events (severe)^a^ | -10.8 | 4.58 | -20.0 to -1.53 | -2.35 | 0.023 |
| Titration (faster) | 0.27 | 3.37 | -6.52 to 7.07 | 0.082 | 0.93 |
| Concomitant valproate use | 2.25 | 3.51 | -4.83 to 9.34 | 0.64 | 0.52 |
| CI, confidence interval; SE, standard error | |  |  |  |  |
| ^a^ The asymptomatic group is the reference. | |  |  |  |  |

| Supplementary Table 3: Multivariate analysis for the date of peak eosinophil count | | | |  |  |
| --- | --- | --- | --- | --- | --- |
|  | Estimate | SE | 95%CI | T | P |
| Inflammatory adverse events (non-severe)^a^ | -8.98 | 4.11 | -17.3 to -0.68 | -2.18 | 0.035 |
| Inflammatory adverse events (severe)^a^ | -12.5 | 5.36 | -23.3 to -1.67 | -2.33 | 0.025 |
| Titration (faster) | -0.88 | 3.94 | -8.83 to 7.07 | -0.22 | 0.82 |
| Concomitant valproate use | 0.98 | 4.11 | -7.30 to 9.27 | 0.24 | 0.81 |
| CI, confidence interval; SE, standard error | |  |  |  |  |
| ^a^ The asymptomatic group is the reference. | |  |  |  |  |

| Supplementary Table 4: Eight cases of serious adverse events with eosinophilia leading to discontinuation of clozapine | | | | | | | | | | | | | | | | | |
| --- | --- | --- | --- | --- | --- | --- | --- | --- | --- | --- | --- | --- | --- | --- | --- | --- | --- |
| Patient | Age　(years) | Sex | BMI (kg/m^2^) | Concomitant use of valproate | Smoking | Clozapine titration rate | Diagnosis | Symptom onset date (day) | Clozapine dose at fever onset (mg) | Fever duration (day) | Maximum body temperature (°C) | Date of peak CRP (day) | Peak CRP value (mg/dL) | Date of first detection of eosinophilia (day) | Date of peak eosinophil count (day) | Peak eosinophil count (/μL) | Date of clozapine discontinuation |
| A | 49 | F | 23.1 | + | - | 0.84 | Pneumonia | 15 | 175 | 7 | 39.5 | 24 | 12.9 | 27 | 35 | 1840 | 24 |
| B | 52 | M | 28.2 | - | - | 0.86 | Liver damage | 12 | 125 | 10 | 39.0 | 19 | 6.3 | 19 | 29 | 2999 | 22 |
| C | 38 | M | 19.1 | + | - | 1 | Renal failure and enteritis | 12 | 125 | 12 | 39.9 | 17 | 21.1 | 21 | 21 | 809 | 22 |
| D | 31 | M | 23.5 | + | - | 1 | Myocarditis | 15 | 150 | 14 | 40.8 | 20 | 18.4 | 27 | 27 | 843 | 24 |
| E | 43 | M | 21.8 | - | - | 1 | Pneumonia | 15 | 150 | 5 | 39.6 | 22 | 7.3 | 23 | 29 | 2780 | 22 |
| F | 41 | M | 23.8 | - | - | 0.81 | Pneumonia and skin rash | 151 | NA | NA | NA | 23 | 4.8 | 30 | 30 | 1308 | 158 |
| G | 36 | M | 34.4 | + | - | 1 | Pneumonia | 10 | 125 | 22 | 39.8 | 18 | 15.7 | 12 | 22 | 1049 | 15 |
| H | 26 | M | 23.5 | + | - | 0.57 | Myocarditis | 15 | 75 | 4 | 39.3 | 19 | 11.4 | 22 | 28 | 1190 | 17 |
| BMI, body mass index; CRP, C-reactive protein; NA, not applicable; M, male; F, female | | | | | | | | | | | | | | | | | |
