# Supplementary material for: Relationship Between Clozapine-Induced Inflammation and Eosinophilia: A Retrospective Cohort Study
Source: Schizophr Bull. 2024 Dec 16;52(1):sbae213. doi: 10.1093/schbul/sbae213 (PMC12809819; doi:10.1093/schbul/sbae213)
Supplement: sbae213_suppl_Supplementary_Figure_S1 [file sbae213_suppl_supplementary_figure_s1.zip › sbae213_suppl_Supplementary_Figures_1_caption.docx]

**Supplementary Figure 1: Trends in eosinophil counts among 17 patients without inflammatory adverse effects.** The left axis shows eosinophil count (/mm³), and the horizontal axis represents the number of days since the initiation of clozapine treatment.
